# *Lactobacillus paracasei* R3 protects against dextran sulfate sodium (DSS)-induced colitis in mice via regulating Th17/Treg cell balance

**DOI:** 10.1186/s12967-021-02943-x

**Published:** 2021-08-18

**Authors:** Juan Huang, Ziyan Yang, Yanyun Li, Xingxing Chai, Yanfang Liang, Bihua Lin, Ziyu Ye, Shaobing Zhang, Zhengping Che, Hailiang Zhang, Xueying Zhang, Zhao Zhang, Tao Chen, Weiqing Yang, Jincheng Zeng

**Affiliations:** 1 Dongguan Key Laboratory of Medical Bioactive Molecular Developmental and Translational Research, Guangdong Provincial Key Laboratory of Medical Molecular Diagnostics, Guangdong Medical University, Dongguan, 523808 China; 2 Department of Pathology, Dongguan Hospital Affiliated To Medical College of Jinan University, Binhaiwan Central Hospital of Dongguan, Dongguan, 523905 China; 3 Research and Development Center, Center of Human Microecology Engineering and Technology of Guangdong Province, Guangzhou, 510535 Guangdong China; 4 Provincial Experimental Teaching Centre, Institute of Laboratory Medicine, Guangdong Provincial Key Laboratory of Medical Molecular Diagnostics, School of Medical Technology, Guangdong Medical University, Dongguan, 523808 China; 5 Department of Clinical Microbiology, Institute of Laboratory Medicine, Guangdong Provincial Key Laboratory of Medical Molecular Diagnostics, School of Medical Technology, Guangdong Medical University, Dongguan, 523808 China; 6 Department of Clinical Laboratories, Xi’an Daxing Hospital, Xi’an 710000, China

**Keywords:** *Lactobacillus paracasei*, Colitis, Dextran sulfate sodium, Treg, Th17, Whole genome sequencing

## Abstract

Inflammatory bowel diseases (IBD), mainly comprising ulcerative colitis (UC) and Crohn's Disease, are most often a polygenic disorder with contributions from the intestinal microbiome, defects in barrier function, and dysregulated host responses to microbial stimulation. Strategies that target the microbiota have emerged as potential therapies and, of these, probiotics have gained the greatest attention. Herein, we isolated a strain of *Lactobacillus paracasei* R3 (*L.p* R3) with strong biofilm formation ability from infant feces. Interestingly, we also found *L.p* R3 strain can ameliorate the general symptoms of murine colitis, alleviate inflammatory cell infiltration and inhibit Th17 while promote Treg function in murine dextran sulfate sodium (DSS)-induced colitis. Overall, this study suggested that *L.p* R3 strain significantly improves the symptoms and the pathological damage of mice with colitis and influences the immune function by regulating Th17/Treg cell balance in DSS-induced colitis in mice.

## Introduction

Inflammatory Bowel Disease (IBD) is a multifactorial chronic disease and mainly includes ulcerative colitis (UC) and Crohn’s disease. It is usually a polygenic disease caused by genetic make-up, barrier function, gut microbiome composition, environmental factor, and mucosal immune response [1]. Although microbiota is not sufficient to create signatures that can differentiate between the disease subtypes or between disease relapse and remission [2], strategies targeting the microbiota have become potential treatments for IBD, among which probiotic treatments have received the most attention.

From discovery to clinical application, probiotics have been developed and explored for many years. The category and mechanism of probiotics are gradually clear, and the clinical application is more and more extensive, and their functions mainly include intestinal protection, immune regulation, antibacterial effect, nutrition, anti-tumor, protecting liver, reducing blood lipid and so on [3–9]. Intestinal probiotics, as a kind of active microorganisms, need to be colonized in human intestinal epithelial cells, and then communicate with the intestinal mucosa layer to play an important role in the metabolic, immune and intestinal health protection function of host individuals [3].

Supplementation of probiotics can be used as an treatment for IBD. However, due to the complex pathogenesis of IBD and the influence of individual probiotics, there are many probiotics in the literature, but few strains are used for actual transformation [2, 10]. Therefore, it is still necessary to provide scientific and detailed evidence for the beneficial effects of probiotics. *Escherichia coli Nissle 1917* is a non-pathogenic probiotic that can relieve clinical symptoms in patients with UC with the same efficacy and safety as mesalazine (MSLZ) [11–13]. The synergistic effect of probiotics and 5-ASA in patients with mild to moderate UC promotes clinical remission. Studies have shown that probiotics could reduce proinflammatory cytokines (such as TNF-α and IL-1β) and increase anti-inflammatory factor (IL-10) via inhibiting TLR4/NF-κB signaling pathway and PI3K/Akt/NF-κB signaling pathway to maintaining UC remission [14]. Administration of *Lactobacillus paracasei* strains also improves immunomodulation and changes the composition of gut microbiota leading to improvement of colitis in mice [15, 16]. Herein, we isolated a strain of *Lactobacillus paracasei* R3 (*L.p* R3) with strong biofilm formation ability from infant feces. Interestingly, we also found *L.p* R3 strain can ameliorate the general symptoms of murine colitis, alleviate inflammatory cell infiltration and inhibit Th17 while promote Treg function in murine DSS-induced colitis.

## Materials and methods

### Probiotics isolation

*L.p* R3 were isolated from baby feces and identified by API 50CH strips (BioMerieux) and 16S rDNA sequence in our laboratory. The strain was grown in Man, Rogosa, Sharpe (MRS) broth (Oxoid, Cambridge, UK) and on MRS agar (Oxoid, Cambridge, UK). Inoculated plates were incubated for 48 h at 35 °C under anaerobic conditions. *L.p* R3 was incubated overnight at 35 °C in MRS broth.

### Detection of growth curve

Preparation of fresh bacterial suspensions for growth curves, gastrointestinal tolerance and biofilm formation experiments. MRS broth was used to activate *L.p* R3 strain, strain cells were collected by centrifugation at 4000×*g* for 10 min, washed twice with PBS, and resuspended at 1.5 × 10^8^ cfu/ml. The fresh suspension was inoculated into 200 ml MRS liquid medium according to 2% (v/v) inoculation and 35 °C culture for 24 h. From 0 to 48 h, sampling every 2 h, measuring the OD_630_ value. Each time point is repeated 3 times, taking the average value. MRS broth was a blank control. And the growth curve was drawn with culture time as horizontal coordinate and absorbance as vertical coordinate.

### Gastrointestinal fluid tolerance

Equipped with artificial intestinal fluid and PH = 2, 3, 4, 7 gastric juice. The prepared artificial intestinal fluid and gastric juice were packed in a small centrifuge tube. The fresh suspension was inoculated into 20 ml MRS liquid medium according to 2% (v/v) inoculation and 35 °C culture for 16 h. Cultures were collected by centrifugation at 4000×*g* for 10 min, washed twice with PBS, discard supernatant. First, the bacterial cells were treated with 4 kinds of gastric juice, 35 °C and 50 r/min, for 3 h, count the number of live bacteria at 30 min, 90 min and 180 min time point. After 3 h of gastric juice treatment, centrifuge to discard gastric juice, then add the same volume of intestinal fluid, 35 °C and 50 r/min, for 3 h, count the number of live bacteria at 270 min and 360 min time point. The concentration of bacterium was determined by colony plate counting method. Each group was set up 3 parallel and repeated 3 times. Survival rate = (number of live bacteria per time point (cfu/ml)/1.5 × 10^8^ cfu/ml) × 100%.

### Biofilm formation

Semi-quantitative determination of biofilm formation ability of strain by micro-pore plate.The fresh suspension was inoculated into 200 ml MRS liquid medium according to 2% (v/v) inoculation, mix well, add to 96 hole plate, each hole 200 μl, 35 °C static culture for 48 h, determine the OD_630_ value by enzyme labeling instrument, recorded as A1, MRS broth was a blank control. Discard excess medium, washed twice with PBS to remove residual media and surface plankton bacteria, with 200 μl methanol fixed for 10 min in each hole, natural drying, with 100 μl 1% crystal violet solution to dye 30 min, washed twice with PBS to remove excess dyes. When dried naturally, 200 μl 95% of ethanol were added to each hole to dissolve the crystal violet completely. The OD_630_ value was measured, recorded as A2. Each group was set up 3 parallel and repeated 3 times. The adhesion rate is used to express the film forming ability, recorded as B, B = A2 − A2C/A1 − A1C, A1C and A2C are the blank control absorbance value when measuring A1 and A2, respectively. The B < 0.1 is non-adhesion, 0.1 ≤ B < 0.5 low-level film formation, 0.5 ≤ B < 1 medium-level film formation and B ≥1 strong adhesion film formation.

### Whole genome sequencing analysis

Sequencing and bioinformatics analysis commissioned by Guangdong Longsee Biomedical Co., Ltd. Using a IIIumina platform, the operation is mainly divided into five steps: extraction of genome DNA, library preparation, high-throughput sequencing, genome assembly, genome fine mapping and biological information analysis (including three aspects of gene prediction, gene annotation and genome comparative analysis). In gene function annotation analysis, a total of 11 databases were used, including ARDB, CARD, CAZY, GO, COG, KEGG, T3SS, NR, DBCAN, SWISSPROT and IPR.

### Safety evaluation of *L.p* R3 strain

Acute toxicity test: 12 Balb/c mice, after one week of adaptive feeding, randomly divided into two groups including control group and *L.p* R3 group, six mice per group. MRS broth was used to activate *L.p* R3 strain, strain cells were collected by centrifugation at 4000×*g* for 10 min, washed twice with PBS, and resuspended at 1 × 10^12^ cfu/ml. The experimental group was given 200 μl of bacterial suspension each time, and the control group was given 200 μl of normal saline each time, once a day, for continuous intragastric administration for 30 days. Record the general signs of the mice daily, such as movement, hair, weight, death or not. On the 31st day, Cervical dislocation method was used to kill and dissect mice to take their organs. To observe the morphological changes of heart, liver, spleen, lung and kidney, and to measure organ quality, Calculate organ index = m1/m2 [m1 is organ quality (g), m2 was weight quality in mice (g)].

### Design of animal experiments

Six-week-old C57BL/6 mice (weighing 20.0 + 2.0 g, from Guangdong Medical Laboratory Animal Center, Foshan, Guangdong Province, China) were housed in plastic cages (at 22 ± 2 °C and 50 ~ 60% relative humidity with a 12 h dark/12 h light cycle) with free access to diet and water. Acute colitis was induced by administering 3% (w/v) DSS (molecular mass 36,000–50,000 Da; MP Biochemicals) in drinking water. The concentrations of *L.p* R3 strain and mesalazine (MSLZ) used in the experiment were 1.0 × 10^9^ CFU/ml and 52 mg/ml, respectively.C57BL/6 mice were randomly divided into five groups (n = 6), including Normal group, DSS group, MSLZ group, *L.p* R3 group and *L.p* R3 combined with MSLZ group (*L.p* R3 + MSLZ group). For 14 days, each mouse was given 200 μl/d by gavage at a fixed daily time. From 8 to 14 days, the normal group was free to drink double distilled water, and the other four groups of mice were free to drink 3% DSS solution for 7 days to establish an acute UC model. Observe and record the daily condition of mice during the test. After the experiment, serum and colon samples from mice were collected for study.

### Determination of disease activity index (DAI)

The disease activity index (DAI) was determined by scoring the degree of weight loss, stool stiffness, and the presence of occult or whole blood (Table 1). Occult blood in faecals was evaluated with a Faecal Occult Blood Test Kit (Jiancheng Biotech, Nanjing, People’s Republic of China).

**Table 1:** Disease activity index score

Score	Weight loss	Stool cinsistency	Gross bleeding
0	None	Normal	Negative
1	1–5%		
2	6–10%	Losse stool	Haemoccult
3	11–15%		
4	> 15%	Diarrhea	Bleeding(visible)

The disease activity index = (combined score of weight loss, stool consistency and bleeding)/3

### Histological assessment

Colon length was measured as described above to assess colon shortening. 1 cm distal colon was fixed with PBS-buffered 10% (v/v) formalin buffer for 48 h, then embedded in paraffin, cut into 5 μm thick sections, hematoxylin and eosin (HE) staining and inspection with light microscope. A blinded histopathologic analysis was used to score the stage of colitis based on morphological criteria Ringed (Table 2). Randomly select 3 sections of each sample and examine 10 random fields (magnification 100×) in each section. The average value for each part is calculated by the average level.

**Table 2:** Histological disease scoring system

Score	Histological features
0	Normal colonic mucosa
1	Loss of one-third of the crypts
2	Loss of two-third of the crypts
3	The lamina propria is covered with a single lay inflammatory cell infiltration is present
4	Erosions and marked inflammatory cell infiltra

### Immunohistochemistry

Immunohistochemistry was performed on 4 um thick paraffinembedded sections of the colon. The with incubated were sections anti-Foxp3 (1:200), anti-CD25 (1:200), anti-IL-17A (1:20 0) at 4 °C overnight. VECTASTAIN ABC Kit (Vector, CA) was used as secondary antibody. Immunoreactivity was analyzed with a microscope (Olympus AX70, Olympus Optical Corp., Tokyo, Japan). The total number of CD25, Foxp3, IL-17A and IL-10 positve cells in each of four nonadjacent fields of view was counted per colon sample (magnification, 100×).

### Western blot

The tissue was homogenized with lysis buffer, an equal amount of protein (50 μg) was analyzed by 12% SDS–polyacrylamide gel electrophoresis, and the blotted membrane was blocked with 5% BSA in TBST for 60 min at room temperature, anti-Foxp3 (1:1000, Abcam), anti-CD25 (1:1000, Affinity Biosciences), anti-IL-17A (1:1000, Affinity Biosciences), anti-L-10 (1:1000, bs-6761R, Bioss) and FN-γ (1:1000, Affinity Biosciences) antibody overnight at 4 °C. GAPDH (1:1000.Sigma) was used as a loading control. The HRP-conjugated anti-rabbit or anti-mouse IgG (1:2000, Cell Signaling) was used as a secondary antibody and the membrane was developed using an enhanced chemiluminescence system (Amersham Pharmacia Biotech).

### Measurement of cytokine

Cytokines such as IL-10, IFN-γ, IL-17A and IL-17F were measured in duplicate with Multi-Analyte Flow Assay Kit (Biolegend).

### Statistical analysis

Figures and statistics were performed using Prism 7.0 a software (Graphpad Inc.). All data represent Mean ± SEM. Data that was not successfully distributed was log converted and evaluated for distribution. Using Student’s t-test or ANOVA with Tukey test to determine significant differences between two groups or analyze significant differences between multiple test groups.

## Results

### Biological properties of *L.p* R3

**Fig. 1:**
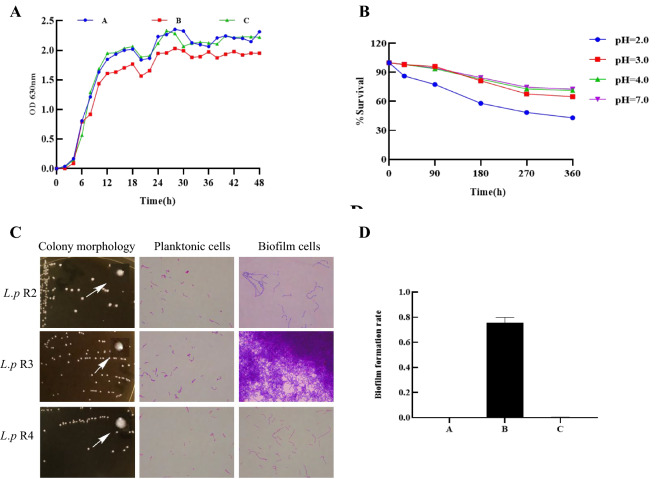
Biological properties of *L.p* R3 strain. **A** Growth curves of *L.p* R2, *L.p* R3 and *L.p* R4 strains were detected. **B** Gastrointestinal tract tolerance of *L.p* R3 strain were examined. **C**, **D** Biofilm formation ability of *L.p* R2, *L.p* R3 and *L.p* R4 strains were measured

*L.p* R3 were isolated from infant feces and identified by API 50CH Biochemical reaction system, named and preserved in our laboratory. The dynamic growth curve in vitro showed that *L.p* R3 strain had a logarithmic phase at 4 h ~ 18 h and then entered the stationary phase, no apparent death phase (Fig. 1A). Under successive effects of artificial gastric and intestinal fluids, the survival rate of *L.p* R3 was above 90% and 70% in PH = 3.0, 4.0 and 7.0 gastric and intestinal fluids, respectively. The survival rate of *L.p* R3 reached (58.76 ± 1.32)% and (43.08 ± 0.27)% after 3 h in PH 2.0 artificial gastric juice and intestinal fluids treatment (Fig. 1B). These results indicate that *L.p* R3 has good tolerance to gastrointestinal fluid and can survive and maintain activity under adverse gastrointestinal conditions. Biofilm formation rate of *L.p* R3 strain was more than 0.6 in the experiment of semi-quantitative determination of biofilm formation ability, showed that the strain had better ability of biofilm formation (Fig. 1C, D).

### Whole genome sequencing analysis of *L.p* R3

**Fig. 2:**
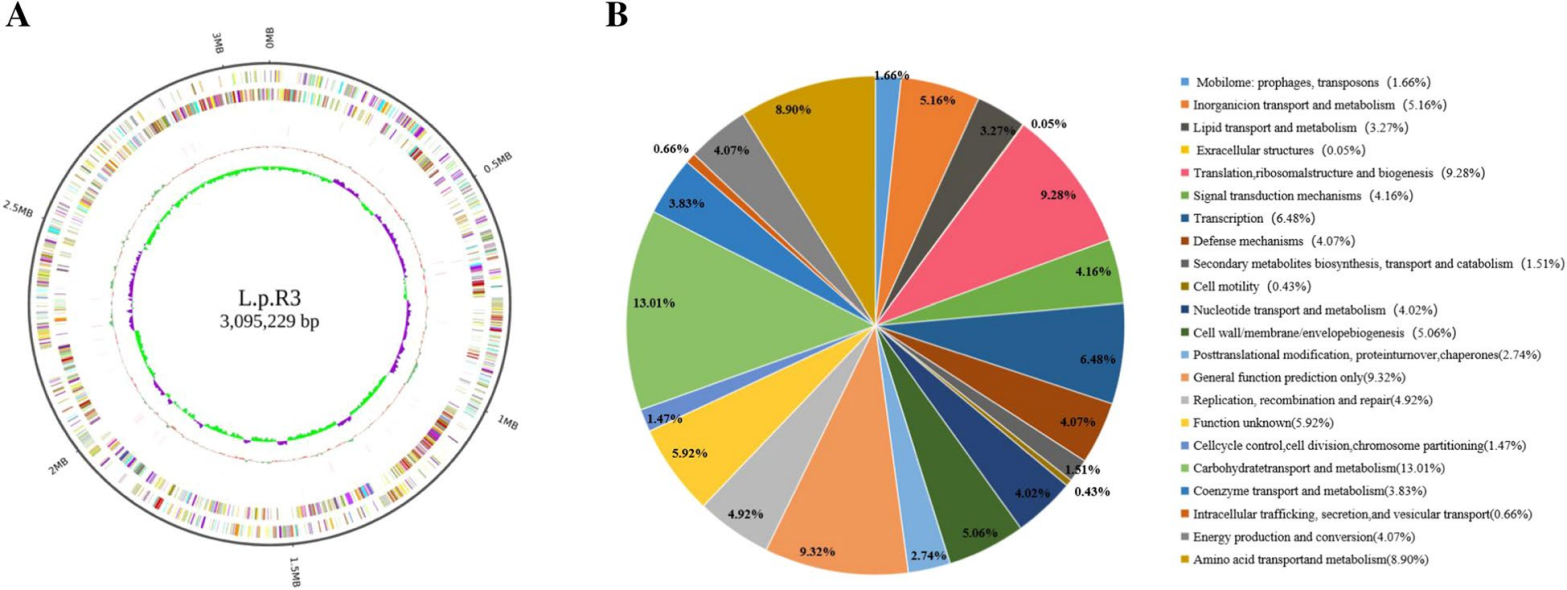
whole genome sequencing analysis of L.p R3 strain. **A** The genome cycle graph of *L.p* R3. **B** COG function classification

To look for other possible characteristics of *L.p* R3, its entire genome was sequenced (Fig. 2 A). The main features of *L.p* R3 genome are reported in Table 3.The assembling of reads generated 92 contigs, giving a genome size of 3.095 Mb with a GC content of 46.17%. Using multiple databases to analyze the gene function of the strain, gene prediction and annotation indicated the total number of 3171 predicted protein-coding sequences (CDSs) and showed 60 structural RNAs. The strain had no plasmids on its chromosomes and no virulence genes were detected. It is noteworthy that the strain genes involve antibiotic resistance genes and TXSS systems associated with pathogenic mechanisms of Gram-negative bacteria. However, the similarities between the detected resistance gene locus and the corresponding antibiotic resistance gene sequences were less than 80%. And its did not evidence any acquired resistance gene in *L.p* R3, hence this strain can be considered safe regarding possible transmissible antibiotic resistances. As for the existence of TXSS system, whether the strain has the risk of infection needs further study. COG database annotations were shown in Fig. 2B. The largest part of this subsystem is allocated to the Carbohydrate transport and metabolism (13.01%), General function prediction only (9.32%), Translation, ribosomal structure and biogenesis (9.28%), Amino acid transport and metabolism (8.90%), Transcription (6.48%), Inorganic ion transport and metabolism (5.16%) and Cell wall/membrane/envelope biogenesis (5.06%), respectively. It is noteworthy that the strain contains proteins related to Lipid transport and metabolism (3.27%) and Mobilome: prophages, transposons (1.66%).

**Table 3:** Genome feature of L.p R3

Attribute	Values
Genome size	3,095,229 bp
G + C content (%)	46.17
Contig N50	89,515 bp
Contig L50	11
Number of contigs	92
Number of protein conding sequence (CDSs)	3171
Number of rRNAs	3
Number of tRNAs	57

### Safety evaluation of *L.p* R3

**Fig. 3:**
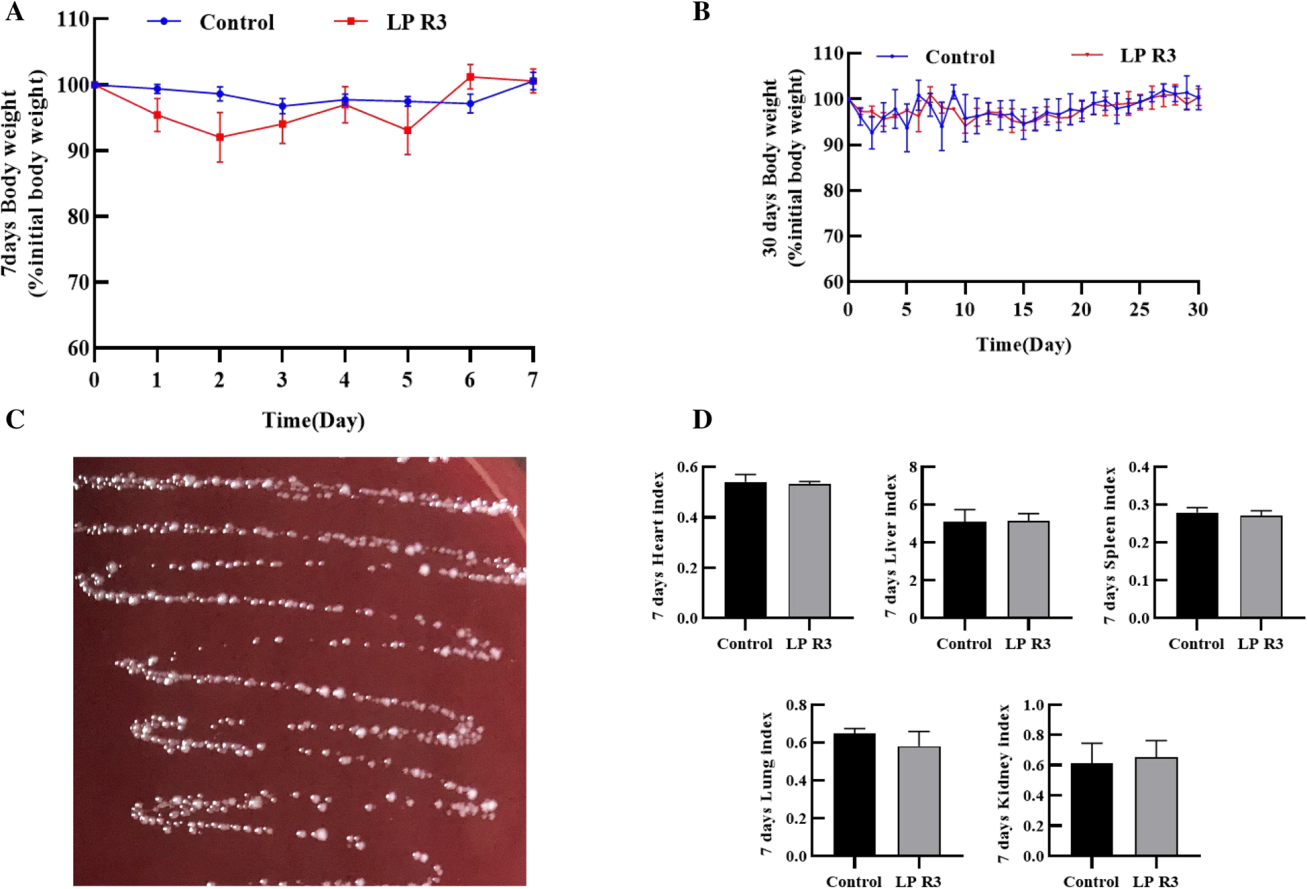
Safety evaluation of *L.p* R3. **A** Weight changes in mice fed for 7 days. **B** Weight changes in mice fed for 30 days. **C** Experimental results of hemolysis. **D** Organ index of mice

*L.p* R3 did not produce hemolysis using colombian blood agar plate by anaerobic culture for 48 h (Fig. 3). By feeding *L.p* R3 (concentration of 1 × 10^12^ cfu/ml) to mice for 7 day and 30 day, there was no significant difference in body weight, organ index, and blood glucose compared with the control group (Fig. 3). Experimental results showed that the activity and mental state (the luster of the eyes) of mice were normal, eating and drinking water were stable, the fur was bright, the fecal state was normal, no death occurred and no lesions were found in heart, liver, spleen, lung and kidney organ. And Body weight and major organ index of mice were not significantly different compared with the control group (p > 0.05) (Fig. 3). The results suggest that the strain had no risk of infection and did not affected the normal physiology of mice.

### *Lactobacillus paracasei (L.p)* R3 strain ameliorates the general symptoms of murine colitis

**Fig. 4:**
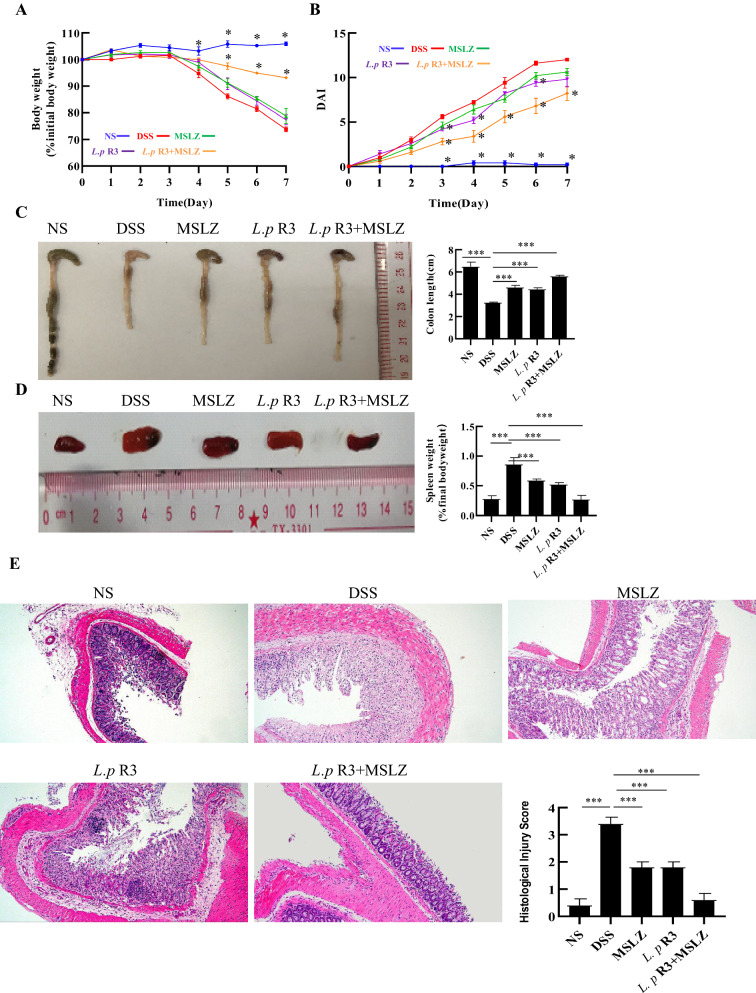
*L.p* R3 strain ameliorates the general symptoms of murine colitis. **A** Percentage change in body weight of mice after induction of colitis. **B** Daily disease activity index (DAI) of mice after induction of colitis. **C** Representative images showing the colon lengths on days 14. **D** Representative images of the spleen and SI on days 14. **E** Histological analysis of the severity of colon sections in DSS-induced colitis mouse model. Colon tissue was paraffin-embedded and stained with hematoxylin and eosin stain (HE stain). Cellular infiltration, crypt distortion, and goblet cell loss were analyzed on days 14. HE stain scale: 100 mm. n = 6, *p < 0.05

In DSS-induced UC model, normal group mice showed normal activity, mental state, normal defecation, bright hair and normal weight gain. And other four groups mice began to develop different degrees of UC symptoms on the third day of free drinking of 3% DSS solution. These results show that the model is successful. Especially, the symptoms of mice in the DSS group are becoming more and more serious, with poor spirit, obvious blood stool and hair removal symptoms. Compared with the treatment group,the weight ratio of the DSS group decreased significantly (p < 0.05) (Fig. 4A). The DAI score, which is an indicator of the severity of colitis, including the extent of body weight loss, haemoccult positivity or gross bleeding and stool consistency, was evaluated for each animal (Table 1). As shown in Fig. 4B, according to normal group, the order of DAI score from largest to smallest is DSS group, MSLZ group, *L.p* R3 group and *L.p* R3 + MSLZ group. DSS group began to show significantly higher DAI values compared to the control group (p < 0.05). The DAI values of MSLZ + *L.p* R3 groups were significantly lower than DSS group (p < 0.05). The results suggesting that *L.p* R3 can alleviate weight loss and UC symptoms in mice caused by DSS.

### *Lactobacillus paracasei *(*L.p*) R3 strain alleviates inflammatory cell infiltration in murine DSS-induced colitis

We studied the colon of experimental mice and the results were shown in Fig. 4C, E. During colonic inflammation, the length of the mouse colon is shortened by ulceration.This is accompanied by adhesions of colonic tissue. In DSS group, colon length was significantly shorter than in the control group (p < 0.05). The colon length was recovered to different degrees in the three treated-groups compared with DSS group, and the recovery was most obvious in *L.p* R3 + MSLZ group (p < 0.001). In further colon histopathological experiments, mice colonic structure damage, glands incomplete, a large number of inflammatory cell infiltration, typical inflammatory changes in DSS group. And the histological injury score of DSS group was significantly higher than that of other four groups (p < 0.001). While the *L.p* R3 administered mice exhibited significantly less histological damage. There was less loss of crypt and goblet cells and less infiltration of various immune cells in the *L.p* R3 administered mice (Fig. 4E). Similarly, the systemic inflammatory response, assessed by splenomegaly, is shown in Fig. 4D. Spleen index were also significantly higher in DSS mice than in controls (*p* < 0.05). In mice fed with *L.p* R3, the mean spleen index was lower than in DSS animals (*p* < 0.05) and more similar to control animals. The results confirmed that the *L.p* R3 strain can relieve inflammation and pathological damage of the colon by DSS-induced.

### *Lactobacillus paracasei (L.p)* R3 strain reverses peripheral blood inflammatory cytokines IL-10/IL-17A ratio in murine DSS-induced colitis

**Fig. 5:**
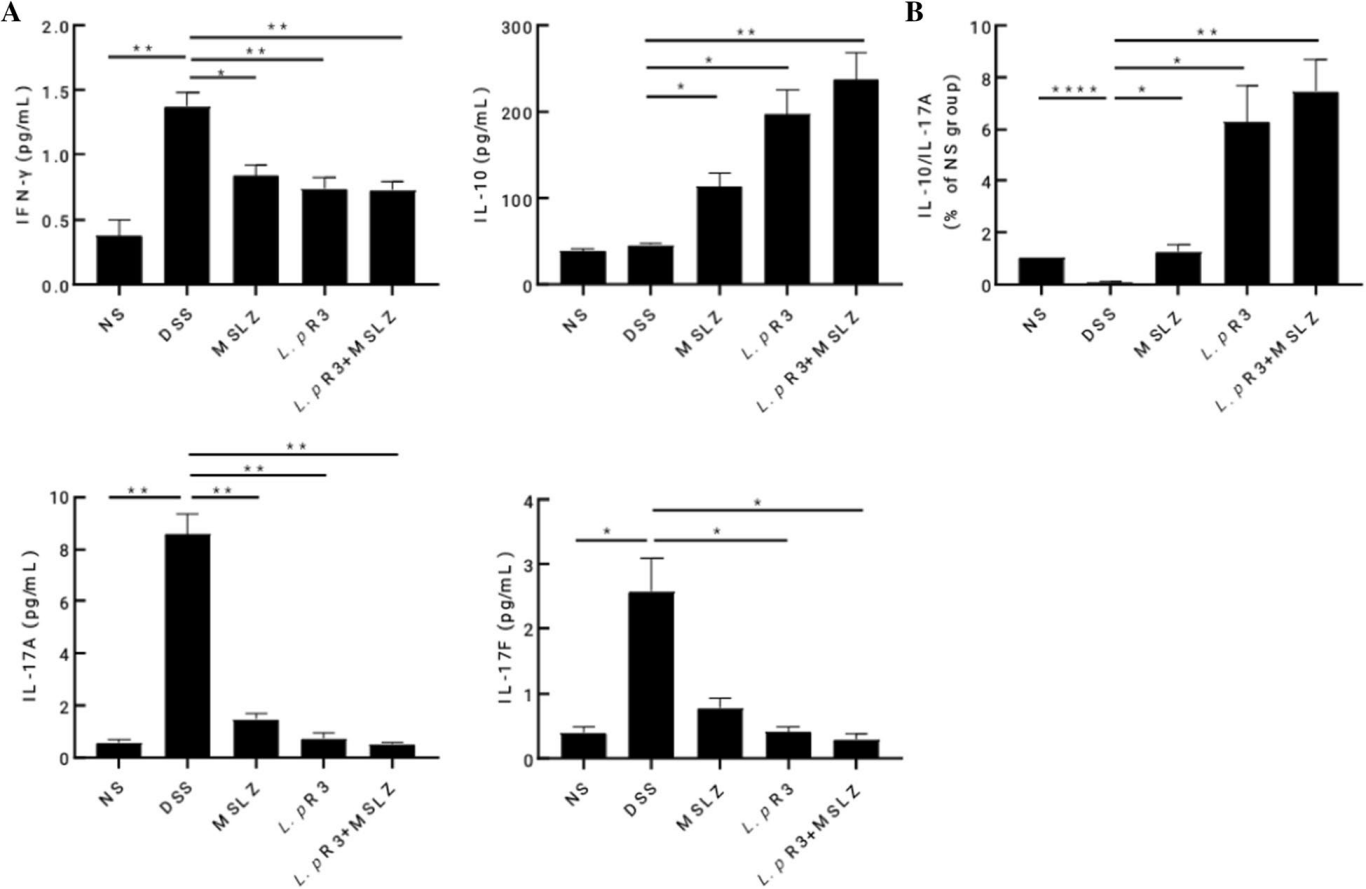
Detection of serum immune cytokines by liquid-phase chip method. **A** The levels of IFN-γ, IL-17A, IL-17F and IL-10 in peripheral blood. **B** The value of IL-17A/IL-10 ratio in peripheral serum of each group mice.*p < 0.05

Among the immune cells involved, specific T cell lineages play an important role in influencing colonic inflammation and pathological responses. In particular, Th1 (producing IFN-γ), Th17 (producing IL-17A and IL-17F) and Treg (producing IL-10) have been reported to be associated with Crohn’s Disease in humans and DSS-induced colitis in mice [17, 18]. Therefore, in order to further evaluate the regulatory effect of *L.p* R3 strain on colonic inflammation, the levels of peripheral serum IFN-γ, IL-10, IL-17A and IL-17F were detected by liquid-phase chip method. As shown in the Fig. 5A, compared with the normal group, the peripheral blood IFN-γ, IL-17A, and IL-17F levels in the DSS group were significantly increased (p < 0.05), while there was no difference in the peripheral blood IL-10 levels between the normal group and the DSS group. Interestingly, after treatment with *L.p* R3 or/and MSLZ, the peripheral blood IFN-γ, IL-17A, and IL-17F levels were significantly decreased (p < 0.05), but blood IL-10 levels were significantly increased (p < 0.05) in the DSS-induced colitis mice. In addition, we set the value of IL-10/IL-17A ratio of the normal group to 1, and then compare the IL-10/IL-17A values of each group to evaluate the changes in the balance of Treg/Th17 cells in mice after treatment. The results showed that the value IIL-10/L-17A ratio of the DSS group was significantly lower than that of the normal group (p < 0.05) (Fig. 5B). This suggests that IL-17A-mediated inflammation (Th17 cells) may play a leading role in DSS-induced intestinal inflammation. However, after treatment with *L.p* R3 or/and MSLZ, the anti-inflammatory factor IL-10 levels were increased and the value of IL-10/IL-17A ratio was significantly increased (p < 0.05) in the DSS-induced colitis mice (Fig. 5B). These results suggest that *L.p* R3 strain can reverses peripheral blood inflammatory cytokines IL-10/IL-17A ratio.

### *Lactobacillus paracasei* (*L.p*) R3 strain inhibits the expression of IFN-γ and IL-17A, while promotes the expression of CD25, Foxp3 and IL-10 in murine DSS-induced colitis tissue

**Fig. 6:**
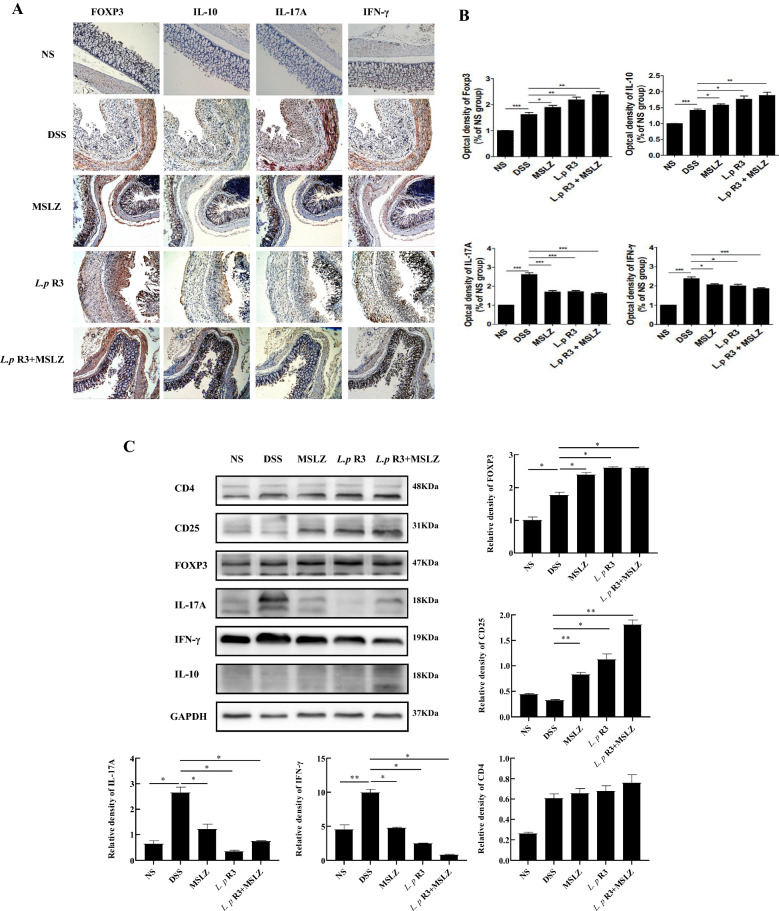
*L.p* R3 attenuates colonic inflammatory responses but induces Treg responses in DSS-induced experimental murine colitis. **A** The expression of Foxp3, IL-10, IL-17A and IFN-γ were detected in the colon by the immunohistochemical method. **B** Pathological score of Foxp3, IL-10, IL-17A and IFN-γ expression in colon tissue. **C** Evaluate the expression of CD4, CD25, Foxp3, IL-17A, IFN-γ, IL-10 in colon tissue by Western blot. *p < 0.05; **p < 0.01; ***p < 0.001

The above results suggest that *L.p* R3 intervention can regulate the levels of peripheral blood cytokines IL-10, IFN-γ and IL-17A. As far as we know, IL-17A is mainly produced by Th17 cells. Treg cells are characterized by the expression of Foxp3, CD25 and CD4, and secreting IL-10. Th1 cells are characterized by producing IFN-γ. Therefore, in order to further determine the level of inflammatory cytokines in pathological tissues and whether these cytokines are related to local tissue-specific T cells, the expression of Foxp3, IL-10, IL-17A and IFN-γ were detected by immunohistochemistry and the expression of CD4, CD25, Foxp3, IL-10, IL-17A and IFN-γ were examined by Western blotting in colonic tissues of mice. As shown in the Fig. 6A, B, compared with the normal group, the expression of Foxp3, IL-10, IL-17A and IFN-γ in the DSS group was significantly increased (p < 0.05). Interestingly, the expression of IFN-γ and IL-17A was significantly reduced (p < 0.05), but the expression of Foxp3 and IL-10 was significantly increased after treatment with *L.p* R3 or/and MSLZ (Fig. 6A, B). In addition, the Western blotting results further showed that the expression of CD4, CD25, Foxp3, IL-17A and IFN-γ was significantly increased in DSS-induced mice (Fig. 6C). The expression of IFN-γ and IL-17A was significantly reduced (p < 0.05), but the expression of CD25 and Foxp3 was significantly increased after treatment with *L.p* R3 or/and MSLZ (Fig. 6C). Therefore, *L.p* R3 strain inhibits the expression of IFN-γ and IL-17A, while promotes the expression of CD25, Foxp3 and IL-10 in murine DSS-induced colitis tissue. These results suggest that Treg/Th17 cell imbalance in the intestinal inflammation caused by DSS was restored after *L.p* R3 strain treatment.

## Discussion

As a member of the traditional probiotics, the *L.p* R3 strain was isolated from infant feces by our team. We studied the survival ability of this strain in vitro. The dynamic growth curve in vitro showed that *L.p* R3 strain had a logarithmic phase at 4 h ~ 18 h and then entered the stationary phase, no apparent death phase. This strain has good characteristics with gastrointestinal fluid tolerance and strong biofilm formation ability. Probiotics are often used as active microorganisms, which need to withstand the persecution of gastrointestinal fluid and reach intestinal colonization with a high number of live bacteria, and to play a role in promoting the health of the body [19]. Colonization is the premise of growth and development of probiotics and a physiological function under adhesion [19]. The colonized bacteria can form biofilm on the surface of adhesion membrane to prevent the invasion of foreign bacteria and protect the health of intestinal mucosa. Therefore, acid tolerance, bile salt tolerance and intestinal mucosal adhesion all used as criteria for screening probiotics.

*Lactobacillus paracasei* (*L.p)* as an important member of Lactobacillus, is Gram-positive bacterium widely found in human oral cavity, intestinal tract and fermented foods. *L.p* can regulate the balance of intestinal flora, enhance human immunity and anti-tumor effects [20–22]. It has been used in food, health care and industrial production, and has received extensive attention at home and abroad. So far, some Lactobacillus members have been found to have anti-inflammatory effects in IBD diseases. *Lactobacillus paracasei subsp. paracasei* NTU 101 (NTU 101) is a multifunctional strain that has been shown in previous studies to possess anti-inflammatory properties and to exert a modulatory effect on intestinal bacteria associated with certain pathogenic mechanisms of IBD [15]. NTU 101 could reduce oxidative stress and the inflammatory response in DSS-treated mice by enhancing the antioxidative capacity of total antioxidants capacity (GR, GSH, CAT, SOD, MDA), and hindering the secretion of proin-inflammatory cytokines (such as TNF-α, IL-6, IFN-γ, and IL-12) [15]. *Lactobacillus paracasei ssp. paracasei* YBJ01 (LPSP-YBJ01) reduced d-galactose-induced oxidation in male Kuming mice. Strain LPSP-YBJ01 significantly increased serum superoxide dismutase (SOD), glutathione peroxidase, and total-antioxidant capability, and inhibited generation of malondialdehyde in a dose-dependent manner [23]. In addition, LPSP-YBJ01 also increased the hepatic and splenic protein expressions of some antioxidant enzymes such as catalase, Cu/Zn-SOD, and Mn-SOD in mice treated with d-galactose [23].

The safety of probiotic strains is also important, including potential pathogenic virulence factor and resistance. At present, domestic probiotic products only list the names of bacteria, without specific strain names and related genetic background information, but the safety of probiotics is strain-specific [24, 25]. Genomics studies on the probiotic and safety of probiotics are essential to obtain lactic acid bacteria that can be used to develop probiotics [26]. This method can fully reveal the genetic information of lactic acid bacteria, systematically explain the physiological functions and metabolic mechanisms of lactic acid bacteria, lay a foundation for the systematic classification and genetic evolution of lactic acid bacteria, and provide a basis for the selection of excellent bacterial species. In this study, the whole genome sequencing showed that the genome size of *L.p* R3 strain is 3.095 Mb with a GC content of 46.17%, and possesses 3171 protein-coding sequences. COG annotation data of *L.p* R3 shown that the protein coding sequence mainly involves the basic physiological activities of the strain, such as carbohydrate transport and metabolism (13.01%), general function prediction only (9.32%), translation, ribosomal structure and biogenesis (9.28%), amino acid transport and metabolism (8.90%), transcription (6.48%), inorganic ion transport and metabolism (5.16%) and cell wall/membrane/envelope biogenesis (5.06%). It is noteworthy that the strain contains proteins related to lipid transport and metabolism (3.27%). *L.p* R3 may have lipid-lowering potential and is worthy of further study. Fortunately, the strain had no plasmids on its chromosomes and no virulence genes were detected. It was safe for antibiotic resistance gene delivery and virulence gene. To further evaluate the safety of *L.p* R3, we also examined its hemolysis on the blood plate and its risk of infection in mice. The *L.p* R3 strain did not produce a hemolytic ring in vitro, nor did it cause infection to mice fed with high concentrations of live bacteria, nor did it affect the normal physiological activities of mice.

As a probiotic strain, *L.p* R3 has been specially studied in our laboratory for its intestinal protective function. The mouse model of ulcerative colitis was successfully induced by DSS. The treatment of UC model mice by intragastric administration of *L.p* R3 significantly relieved UC symptoms and pathological damage of colon tissue. In our study, the UC model mice had a significant weight loss, poor mental state, hematochezia and hair loss, and their colonic pathology showed mucosal tissue damage and extensive inflammatory cell infiltration. Interestingly, the above-mentioned conditions of colitis mice treated with MSLZ or *L.p* R3 were significantly improved. DAI and histological damage scores were used to quantify the extent of damage in each group mice. The values of two indicators are in the same order, and the order from largest to smallest is DSS group, MSLZ group, *L.p* R3 group and *L.p* R3 + MSLZ group. This study shows that *L.p* R3 has a therapeutic effect on UC, and the combination of probiotics and MSLZ has a better effect.

UC is a refractory inflammatory disease that mainly affects the intestine, and is one of the chronic diseases that endanger human health. Global prevalence is projected to affect up to 30 million individuals by 2025 [27]. The incidence of UC continues to increase, but so far, the specific pathogenesis of UC is unknown. It is generally believed that the pathogenesis of UC involves the results of multiple interactions including genetics, microorganisms, and the body's immune system [28]. In recent years, the pathogenesis of UC has been continuously studied through UC patients and animal models of UC, and it has been found that the occurrence and development of UC are closely related to the balance of Foxp3 + Treg cells and Th17 cells (producing IL-17A and IL-17F) in vivo [29–31]. Treg cells are a subset of T cells with negative immune regulation. Treg cells play an important role in maintaining immune balance and forming peripheral immune tolerance. Treg cells can participate in the inflammatory response in vitro through a cell contact-dependent mechanism. Treg cells can also participate in the pathogenesis of UC by regulating the release of inhibitory cytokines (IL-10, etc.), and their low number or dysfunction can lead to the occurrence of the disease [31–33]. Th17 is a subset of T cells that can secrete IL-17A. IL-17A is a pro-inflammatory cytokine with pro-inflammatory activity and plays different roles in different diseases. Compared with healthy individuals, IL-17A levels in UC patients were significantly increased. Similarly, IL-17A levels in ulcerative colitis mouse models were also significantly increased [34, 35].

In this study, we found that Treg and Th17 cells maintain a balance under normal conditions. However, in an inflammatory state, Th17 cells migrate from the circulatory system to the local inflammation site of the intestine, and are enriched in the inflammation injury site. As a result, the mucosa of the digestive tract is highly activated, which in turn induces the intestinal immune response, increases the release of harmful cytokines, leads to mucosal damage, and compensatory increases in Treg cells. Here we used immunohistochemistry and Western blotting to detect the expression of Th17 and Treg cell markers in colon tissue. The results showed that the expressions of Foxp3, IL-10, IL-17A and IFN-γ in the DSS group and the other three treatment groups increased to varying degrees. Compared with DSS group, *L.p* R3 strain had the same effect as MSLZ. After treatment with L.p R3 strain, the expression of Foxp3 and IL-10 increased, while the expression of IL-17A and IFN-γ decreased (p < 0.05). Similarly, Western blotting results showed that the expressions of CD25, and Foxp3 in the three treatment groups were higher than those in the DSS group, while the expressions of IL-17A and IFN-γ in the treatment group were significantly lower than those in the DSS group (p < 0.05). Obviously, *L.p* R3 can promote the expression of Treg cell markers and inhibit the expression of Th17 cell markers. After treatment with *L.p* R3, the imbalance of Treg/Th17 cells in the inflammation caused by DSS was restored.

Using the detection of mouse serum cytokines to study the anti-inflammatory mechanism of *L.p* R3, the results showed that the levels of IFN-γ, IL-17A, IL-17F and IL-10 in the DSS group increased, except for IL-10, the difference was significant (p < 0.05). Interestingly, the three treatment groups are just the opposite of the model group. The levels of peripheral blood IFN-γ, IL-17A and IL-17F in the three treatment groups were significantly reduced, and IL-10 was significantly increased (p < 0.05). In addition, the value of IL-17A/IL-10 ratio of the DSS group was significantly higher than the other four groups (p < 0.05). This indicates that Th17 cells infiltrate and secrete the pro-inflammatory factor IL-17A during inflammation. With *L.p* R3 or/ and MSLZ treatment, the levels of ant-inflammatory factor IL-10 increased, the value of IL-17A/IL-10 ratio was reversed. To further confirm that *L.p* R3 has a good therapeutic effect on UC by regulating Th17/Treg cells balance.

Therefore, we speculate that supplementing *Lp R3* can improve the imbalance of intestinal microbes, down-regulate the activity of Th17 cells, reduce the secretion of pro-inflammatory cytokines, induce the production of Treg cells, and achieve a new balance of intestinal inflammation and anti-inflammatory cytokines. Thereby improving intestinal inflammation and mucosal damage, which may be one of the important mechanisms of *Lp R3* in the treatment of UC. In this study, the therapeutic effect of *L.p R3* is similar to that of mesalazine, and the combined treatment effect is better, which provides a new choice for clinical treatment.

## Conclusions

In summary, *L.p* R3 can significantly improve the symptoms and pathological damage of colitis mice by regulating the balance of Treg/Th17 cells in DSS-induced mouse colitis, and affect immune function. In addition, *L.p* R3 has the advantages of high safety, good gastrointestinal fluid tolerance, and strong biofilm formation ability, which provides a good foundation for commercial production. However, considering the complexity between humans and experimental animals, whether the clinical application of *L.p* R3 bacteria can achieve the same therapeutic effect requires further research.

## Data Availability

The authors confirm that the data supporting the findings of this study are available within the article.

## Abbreviations

*L.p*: *Lactobacillus paracasei*
UC: Ulcerative colitis
IBD: Inflammatory bowel diseases
DSS: Dextran sulfate sodium
DAI: Disease activity index
MSLZ: Mesalazine
